# Low PHASES-Estimated Rupture Risk Among Patients Presenting with Ruptured Intracranial Aneurysms: A Retrospective Single-Centre Observational Study

**DOI:** 10.3390/jcm15207793

**Published:** 2026-10-09

**Authors:** Mateusz Krakowiak, Bartłomiej Kulesza, Cezary Grochowski, Barbara Maria Myszkowska, Damian Palus, Agnieszka Brzozowska, Paweł Sokal

**Affiliations:** 1 Department of Neurosurgery, Jan Biziel University Hospital No. 2 in Bydgoszcz, 85-168 Bydgoszcz, Poland; mateusz.krakowiak@gmail.com (M.K.); damian.palus@gumed.edu.pl (D.P.); 2 Department of Neurosurgery, John Paul II Catholic University of Lublin, 20-950 Lublin, Poland; bartlomiej.kulesza@kul.pl (B.K.); cezary.grochowski@o2.pl (C.G.); 3 Department of Plastic Surgery, Lublin Oncology Center, 20-090 Lublin, Poland; 4 Department of Information Technology and Medical Statistics with E-Health Laboratory, Faculty of Medical Sciences, Medical University of Lublin, 20-059 Lublin, Poland; 5 Department of Neurosurgery, Collegium Medicum in Bydgoszcz, 85-168 Bydgoszcz, Poland; pawel.sokal@cm.umk.pl

**Keywords:** intracranial aneurysm, aneurysmal subarachnoid haemorrhage, PHASES score, aneurysm rupture, risk assessment, small aneurysm

## Abstract

**Background/Objectives:** The PHASES score is widely used to estimate the 5-year rupture risk of unruptured intracranial aneurysms, yet many patients present with aneurysmal subarachnoid haemorrhage despite low calculated risk. This study aimed to characterise the demographic and aneurysm-related profile of patients with ruptured aneurysms classified as low risk by PHASES. **Methods:** This retrospective single-centre observational study included 243 consecutive patients with radiologically confirmed ruptured intracranial aneurysms treated between 2013 and 2021. PHASES scores and estimated 5-year rupture risks were calculated retrospectively. Age, sex, arterial hypertension, aneurysm size, location, and multiplicity were analysed using non-parametric statistical methods. **Results:** The mean PHASES score was 5.16 ± 3.43 points. An estimated 5-year rupture risk below 1% was assigned to 125 patients (51.44%), and 54 patients (22.22%) had PHASES scores of 0–2. Ruptured aneurysms measuring ≤7 mm occurred in 147 patients (60.49%), and 210 patients (86.42%) were younger than 70 years. Internal carotid artery aneurysms were the most frequent overall, whereas anterior communicating artery aneurysms had the smallest median diameter while remaining among the most common rupture sites. Arterial hypertension was associated with larger aneurysm diameter. **Conclusions:** Low PHASES-estimated risks were common in this cohort of patients presenting with aneurysm rupture. Because the study included no comparison group with unruptured aneurysms, it cannot assess PHASES calibration or predictive performance. PHASES should therefore be used as a population-based decision-support tool rather than a stand-alone predictor of individual rupture risk, with morphology, imaging findings, documented growth, and patient-specific factors incorporated into clinical assessment.

## 1. Introduction

Intracranial aneurysms (IAs) are detected in approximately 3–5% of the adult population, mainly owing to the widespread use of computed tomography angiography and magnetic resonance angiography. Although most aneurysms remain asymptomatic, rupture results in aneurysmal subarachnoid haemorrhage (aSAH), one of the most devastating cerebrovascular diseases, with case-fatality rates approaching 35–40% and permanent neurological disability affecting a substantial proportion of survivors. Accurate identification of aneurysms at increased risk of rupture therefore remains a major challenge in contemporary cerebrovascular neurosurgery and vascular neurology [1,2,3,4,5].

Because preventive microsurgical clipping and endovascular treatment are associated with procedure-related morbidity, treatment decisions require the estimated natural history of an aneurysm to be balanced against treatment risk. Several prediction models have therefore been developed to support individualised management, among which the PHASES score remains the most widely used. The PHASES model was derived from individual patient data obtained from six prospective cohort studies including 8382 patients and estimates the 5-year rupture risk using six predictors: population, hypertension, age, aneurysm size, previous subarachnoid haemorrhage, and aneurysm site [6].

Although PHASES has been externally validated and remains a valuable aid for population-based risk estimation, several retrospective studies have questioned its performance in patients who subsequently experience aneurysm rupture.Sturiale et al. demonstrated that 46% of ruptured aneurysms belonged to the group with a predicted 5-year rupture risk below 1% [7], whereas Pagiola et al. reported that almost 80% of patients with aneurysmal subarachnoid haemorrhage had predicted rupture risks below 5% [8]. Similar findings have been confirmed in other retrospective cohorts, suggesting that rupture may occur despite apparently reassuring PHASES estimates [9,10].

Another unresolved issue concerns aneurysm size. Although aneurysm diameter constitutes one of the strongest components of the PHASES algorithm, rupture frequently occurs in lesions that do not meet conventional treatment thresholds. Previous studies have shown that approximately 12% of ruptured aneurysms are microaneurysms (≤3 mm), whereas approximately one-fifth measure ≤5 mm at rupture [11]. Large retrospective cohorts have also reported typical rupture diameters below 7 mm, indicating that aneurysm size alone is insufficient for accurate rupture-risk stratification [12,13].

More recently, increasing attention has focused on factors that are not incorporated into PHASES. Comparative studies evaluating different clinical risk scores, including the Unruptured Intracranial Aneurysm Treatment Score (UIATS), indicate that currently available models should be regarded as complementary decision-support tools rather than stand-alone predictors of aneurysm rupture [14,15,16].

We hypothesised that patients presenting with aneurysmal subarachnoid haemorrhage despite low PHASES scores share distinct demographic and aneurysm-related characteristics that may not be adequately represented within the current PHASES algorithm. This study therefore aimed to characterise the clinical profile of patients with ruptured aneurysms despite low predicted rupture risk according to PHASES.

## 2. Materials and Methods

### 2.1. Study Design and Patient Population

The institutional database contained 387 patient records reviewed for eligibility between 2013 and 2021. After application of the predefined eligibility criteria, 144 records were not included or were excluded, leaving a final analytic cohort of 243 consecutive eligible patients with aneurysmal subarachnoid haemorrhage treated at the Department of Neurosurgery and Neurology, Jan Biziel University Hospital No. 2, Bydgoszcz, Poland.

Inclusion criteria were: (1) radiologically confirmed aneurysmal subarachnoid haemorrhage; (2) identification of a ruptured intracranial aneurysm; (3) aneurysm assessment using three-dimensional rotational angiography; and (4) availability of clinical and imaging information required for PHASES score calculation.

Non-inclusion criteria were: (1) non-aneurysmal subarachnoid haemorrhage and (2) absence of a radiologically confirmed ruptured intracranial aneurysm.

Exclusion criteria were: (1) incomplete or indeterminate radiological data preventing adequate assessment of aneurysm size or location; (2) missing clinical information required for PHASES score calculation; and (3) multiple or anatomically ambiguous aneurysms when the ruptured culprit aneurysm could not be identified with sufficient confidence. Patients with multiple aneurysms were retained when the source of haemorrhage could be identified reliably.

Demographic data, including age, sex, arterial hypertension, aneurysm location, aneurysm size, and the presence of multiple aneurysms, were obtained from electronic medical records and imaging studies. Hypertension was considered present when a diagnosis of arterial hypertension was documented in the available clinical record. All patients underwent three-dimensional rotational angiography, and angiographically evident partial aneurysm thrombosis was assessed during each examination. Patients with multiple aneurysms remained eligible when the haemorrhage-source aneurysm could be identified reliably; annotations referring to an additional aneurysm without SAH denoted an unruptured coexisting aneurysm in a patient who had aSAH and did not exclude that patient from the cohort. The ruptured culprit aneurysm was identified based on the distribution of subarachnoid blood, aneurysm morphology, angiographic findings, and intraoperative findings where available.

The study was conducted in accordance with the Declaration of Helsinki and approved in 2022 by the Bioethics Committee at Nicolaus Copernicus University (protocol code 346/2022). The requirement for written informed consent was waived owing to the retrospective nature of the study.

### 2.2. PHASES Score Assessment

For each patient, the PHASES score was calculated according to the original model proposed by Greving et al. [6]. The score includes six variables: population, hypertension, age, aneurysm size, earlier subarachnoid haemorrhage, and aneurysm site. For the Population component, the cohort was assigned to the European non-Finnish category (0 points), consistent with the study setting in Poland. No earlier SAH from another aneurysm was documented in the available dataset; accordingly, 0 points were assigned for this component in the reconstructed score. For aneurysm site, ICA aneurysms were assigned 0 points, MCA aneurysms 2 points, and ACA/AComA/PComA or posterior-circulation aneurysms 4 points. The anatomical labels used in the database were mapped to these PHASES site categories according to the original score definition.

**Figure 1:**
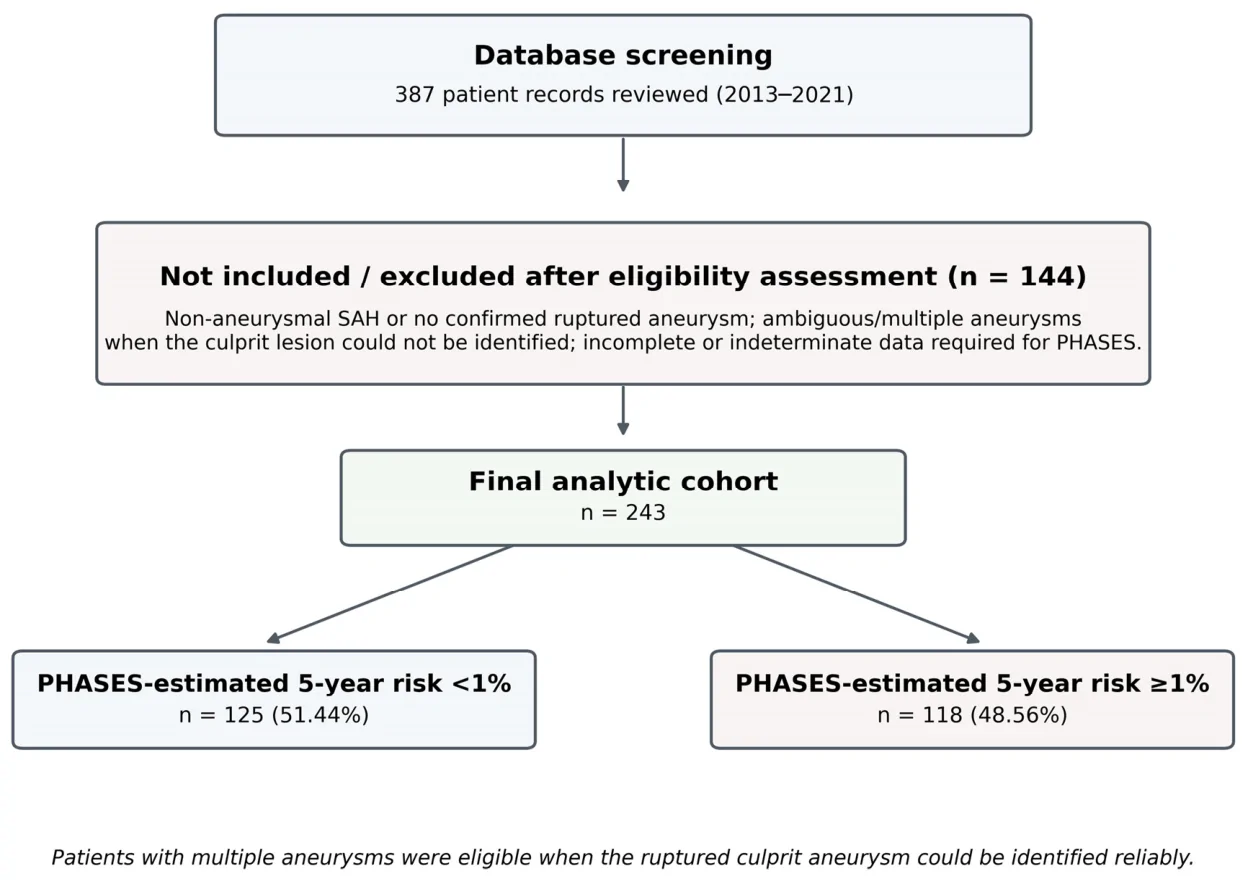
Study flow diagram. A total of 387 patient records were reviewed for eligibility; 243 patients constituted the final analytic cohort after 144 records were not included or excluded according to the predefined eligibility criteria. Patients with multiple aneurysms were eligible when the ruptured culprit aneurysm could be identified reliably. Abbreviations: *n*, number of patients; SAH, subarachnoid haemorrhage; PHASES, Population, Hypertension, Age, Size, Earlier subarachnoid haemorrhage, and Site.

To better evaluate the limitations of the PHASES score, patients were additionally stratified into three score categories (0–2, 3–5, and ≥6 points) as well as according to the estimated 5-year rupture risk (<1%, 1–2%, 2–5%, and ≥5%). The study flow is shown in Figure 1.

### 2.3. Statistical Analysis

Continuous variables are presented as mean ± standard deviation (SD) or median with interquartile range (IQR), depending on data distribution, while categorical variables are expressed as numbers and percentages.

The normality of continuous variables was assessed using the Shapiro–Wilk test. Comparisons between two independent groups were performed using the Mann–Whitney U test, whereas comparisons involving more than two groups were performed using the Kruskal–Wallis test. Associations between categorical variables were evaluated using the chi-square test. Correlations between continuous variables were assessed using Spearman’s rank correlation coefficient.

Variables included in the subgroup and association analyses were age, arterial hypertension, aneurysm size, aneurysm location, and aneurysm multiplicity. Statistical significance was defined as a two-sided *p*-value < 0.05. Given the exploratory nature of the analyses, no formal adjustment for multiple comparisons was applied; *p*-values should therefore be interpreted as exploratory. For an additional descriptive within-cohort analysis, the cohort was stratified according to PHASES-estimated 5-year risk <1% versus ≥1%. Because age, hypertension, aneurysm size, and aneurysm site are themselves components of PHASES and therefore contribute directly to the group definition, this comparison was descriptive only and no inferential between-group testing of these PHASES components was performed. Statistical analyses were performed using STATISTICA 13.0 software (TIBCO Software Inc., Palo Alto, CA, USA).

During preparation of the manuscript, ChatGPT (OpenAI, GPT-6) was used for language editing and assistance with figure layout. The authors reviewed and edited all output and took full responsibility for the content of the publication.

## 3. Results

### 3.1. Patient Characteristics

Baseline demographic, clinical, and aneurysm characteristics are summarised in Table 1.

A total of 243 patients with ruptured intracranial aneurysms were included in the analysis. The mean age of the study population was 56.13 ± 13.10 years, with a median age of 57.0 years and a range from 21 to 93 years. The cohort included 85 men (34.98%) and 158 women (65.02%).

Among the 243 patients included in the study, 229 (94.24%) had a single aneurysm, 13 (5.35%) had two aneurysms, and one patient (0.41%) presented with three aneurysms.

The mean maximum aneurysm diameter was 7.85 ± 4.76 mm, with a median of 6.50 mm and a range from 1.5 to 30.0 mm. In women, the mean maximum aneurysm diameter was 8.02 ± 5.25 mm, whereas in men it was 7.54 ± 3.68 mm.

### 3.2. Aneurysm Location

The most common aneurysm location was the internal carotid artery (ICA), observed in 70 patients (28.81%). Aneurysms of the anterior communicating artery (AComA) were identified in 65 patients (26.75%), and middle cerebral artery (MCA) aneurysms in 54 patients (22.22%). Other locations accounted for 37 cases (15.23%), whereas basilar artery aneurysms were found in 17 patients (7.00%).

Aneurysm location differed significantly according to sex (χ^2^ = 20.21; *p* = 0.0005). In men, AComA aneurysms were the most frequent location, accounting for 36 cases (42.35%). In women, ICA aneurysms predominated and were observed in 56 cases (35.44%). MCA aneurysms occurred with similar frequency in men and women, at 20.00% and 23.42%, respectively.

The distribution of the number of aneurysms did not differ significantly between men and women (χ^2^ = 3.69; *p* = 0.16).

### 3.3. Relationship Between Age, Number of Aneurysms and Aneurysm Size

No statistically significant differences in age were observed according to the number of aneurysms (H = 2.25; *p* = 0.33).

The maximum aneurysm diameter did not differ significantly between anatomical locations (H = 7.67; *p* = 0.10). The highest median maximum diameter was observed for basilar artery aneurysms (8.0 mm). The median diameter was 7.0 mm for ICA, MCA and other locations, and 6.0 mm for AComA aneurysms.

No significant correlation was found between age and the number of aneurysms (R = −0.09; *p* = 0.17).

### 3.4. PHASES Score Characteristics

Hypertension was documented in 43 patients (17.70%), while 33 patients (13.58%) were aged 70 years or older. No earlier SAH from another aneurysm was documented in the available dataset used for PHASES reconstruction.

According to the aneurysm-size categories used in PHASES, 122 patients (50.21%) had aneurysms measuring <7.0 mm, 59 patients (24.28%) had aneurysms measuring 7.0–9.9 mm, 51 patients (20.99%) had aneurysms measuring 10.0–19.9 mm, and 11 patients (4.52%) had aneurysms measuring ≥20.0 mm. The <7.0 mm threshold in this paragraph reflects the PHASES size category and is distinct from the descriptive ≤7.0 mm threshold reported below.

According to PHASES location categories, ICA aneurysms, assigned 0 points, were present in 70 patients (28.81%). MCA aneurysms, assigned 2 points, were present in 54 patients (22.22%). Aneurysms assigned to the ACA/AComA/PComA or posterior-circulation category (4 points) were present in 119 patients (48.97%). Table 2 provides the anatomical mapping used for this categorisation, including the composition of the 37 cases reported descriptively as “other locations”.

For clarity, the complete PHASES scoring system, including the point allocation for each component and the corresponding numbers observed in the present cohort, is summarised in Table 3. The mapping of total PHASES scores to estimated 5-year rupture risk is shown in Table 4.

The mean PHASES score was 5.16 ± 3.43 points, with a median of 4.0 points and a range from 0 to 16 points. The 95% confidence interval for the mean PHASES score was 4.72–5.59 points.

The median estimated 5-year rupture risk according to PHASES was 0.9%, with a range from 0.4% to 17.8%. The mean estimated 5-year rupture risk was 2.37 ± 3.46%, with a 95% confidence interval of 1.93–2.80%.

### 3.5. Distribution of PHASES Risk Groups

**Figure 2:**
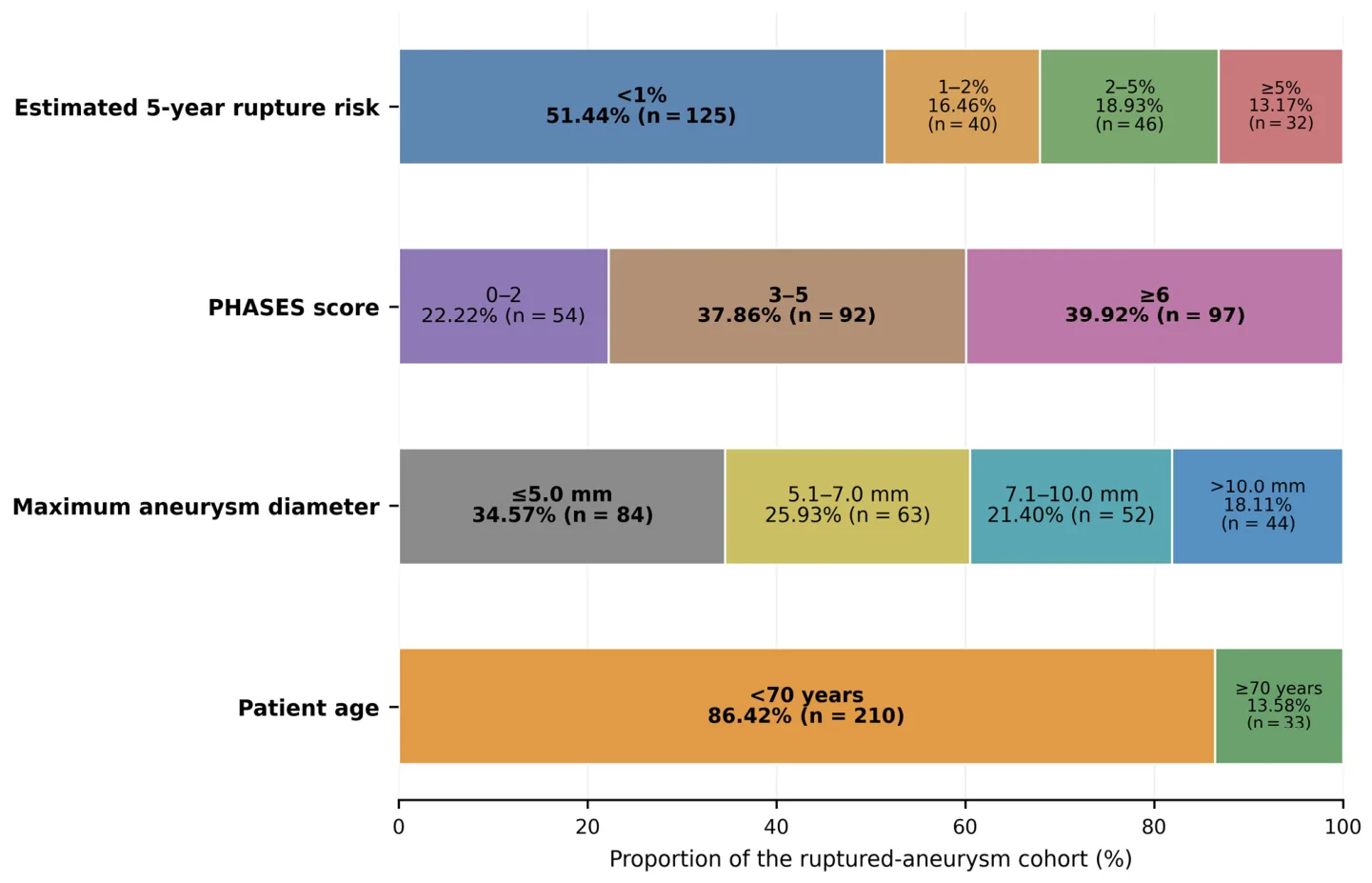
Distribution of PHASES-estimated 5-year rupture risk, PHASES score categories, maximum aneurysm diameter, and patient age in the cohort of 243 patients presenting with ruptured intracranial aneurysms. Percentages represent proportions of the entire cohort; minor discrepancies from 100% may result from rounding. Abbreviations: PHASES, Population, Hypertension, Age, Size, Earlier subarachnoid haemorrhage, and Site; *n*, number of patients.

The distribution of PHASES-estimated risk categories and key cohort characteristics is shown in Figure 2.

When patients were classified according to PHASES score groups, 54 patients (22.22%) had a score of 0–2 points, 92 patients (37.86%) had a score of 3–5 points, and 97 patients (39.92%) had a score of 6 points or more.

Based on the estimated 5-year rupture risk, 125 patients (51.44%) were classified as having a risk below 1%, 40 patients (16.46%) had a risk of 1–2%, 46 patients (18.93%) had a risk of 2–5%, and 32 patients (13.17%) had a risk of 5% or higher.

Aneurysms measuring ≤5.0 mm were observed in 84 patients (34.57%), aneurysms measuring 5.1–7.0 mm in 63 patients (25.93%), aneurysms measuring 7.1–10.0 mm in 52 patients (21.40%), and aneurysms measuring >10.0 mm in 44 patients (18.11%). Overall, 147 patients (60.49%) had ruptured aneurysms measuring ≤7.0 mm. This descriptive ≤7.0 mm threshold includes aneurysms measuring exactly 7.0 mm and therefore differs from the <7.0 mm PHASES category reported above.

### 3.6. Descriptive Comparison of PHASES-Estimated Risk < 1% Versus ≥1%

For additional within-cohort characterisation, the 125 patients with a PHASES-estimated 5-year risk < 1% were descriptively compared with the 118 patients with an estimated risk ≥ 1% (Table 5). The very-low-risk group was younger, had less frequently documented hypertension, and more often had aneurysms in the <7.0 mm PHASES size category. These differences are expected in part because age, hypertension, aneurysm size, and site are components used to calculate PHASES; accordingly, no inferential testing of these variables between PHASES-defined groups was performed.

### 3.7. Age-Related Aneurysm Characteristics

Aneurysms smaller than 7 mm were found in 14 of 33 patients (42.42%) aged 70 years or older and in 108 of 210 patients (51.43%) younger than 70 years. The difference was not statistically significant (χ^2^ = 0.92; *p* = 0.34).

The distribution of aneurysm location did not differ significantly between patients younger than 70 years and those aged 70 years or older (χ^2^ = 0.10; *p* = 0.95).

Using a detailed age-group classification, the largest subgroup consisted of patients aged 60–69 years (*n* = 70; 28.81%). Patients aged 50–59 years accounted for 63 cases (25.93%), those aged 40–49 years for 53 cases (21.81%), patients aged 70 years or older for 33 cases (13.58%), and patients younger than 40 years for 24 cases (9.88%).

No significant correlation was found between age and maximum aneurysm diameter (R = 0.08; *p* = 0.20).

The proportion of aneurysms smaller than 7 mm was highest among patients younger than 40 years, in whom such aneurysms were observed in 17 of 24 cases (70.83%). The corresponding proportions were 58.49% in patients aged 40–49 years, 46.03% in those aged 50–59 years, 44.29% in those aged 60–69 years, and 42.42% in patients aged 70 years or older. These differences were not statistically significant (χ^2^ = 7.76; *p* = 0.11).

### 3.8. Association of Hypertension with Aneurysm Size

Hypertension was also associated with a larger maximum aneurysm diameter. The median maximum aneurysm diameter was 7.0 mm in patients with hypertension and 6.0 mm in patients without hypertension (*p* = 0.006).

## 4. Discussion

The PHASES score remains the most widely accepted clinical tool for estimating the risk of rupture of unruptured intracranial aneurysms. Developed from six prospective cohort studies comprising more than 8000 patients, the model estimates cumulative rupture risk using six readily available clinical and radiological variables [6]. Since its publication, PHASES has been incorporated into international recommendations and is commonly used to support clinical decision-making regarding preventive treatment of unruptured intracranial aneurysms [17].

Despite its widespread acceptance, an increasing number of studies have examined the limitations of applying PHASES to patients who ultimately experience aneurysm rupture. The present study contributes to this discussion by characterising patients in whom rupture occurred despite low retrospectively reconstructed PHASES estimates, while recognising that a ruptured-only cohort cannot establish model miscalibration or underestimation of absolute rupture risk.

The most important finding of our study was that more than half of the patients (51.44%) presented with an estimated 5-year rupture risk below 1% according to the PHASES score despite suffering aneurysmal subarachnoid haemorrhage. Furthermore, nearly one-quarter of patients had PHASES scores of ≤2 points, corresponding to the lowest-risk category. These findings indicate that low PHASES estimates do not necessarily identify patients at negligible individual rupture risk and should not be interpreted as evidence that aneurysm rupture is unlikely on an individual basis.

Our observations are consistent with previous retrospective analyses. Sturiale et al. reported that 46% of ruptured aneurysms belonged to the <1% predicted 5-year rupture-risk category [7]. Similarly, Pagiola et al. demonstrated that almost 80% of patients with aneurysmal subarachnoid haemorrhage had estimated rupture risks below 5% [8]. Other investigators have reached similar conclusions, showing that many ruptured aneurysms would have been classified as low-risk lesions before rupture [7,9,13]. The reproducibility of this pattern across retrospective ruptured-aneurysm cohorts supports caution when applying PHASES to individual treatment decisions, although such studies do not directly assess model calibration in populations with unruptured aneurysms.

One of the most clinically relevant observations in our study concerns aneurysm size. Although aneurysm diameter represents one of the strongest determinants within the PHASES algorithm, 60.49% of ruptured aneurysms measured ≤7 mm. These findings agree with previous reports demonstrating that rupture frequently occurs in relatively small aneurysms. Approximately 12% of ruptured aneurysms have been reported to be microaneurysms (≤3 mm), whereas approximately 21% measured ≤5 mm at rupture [11]. A recent systematic review further demonstrated that morphology-related variables, including aspect ratio, size ratio, and aneurysm irregularity, may improve identification of rupture-prone small aneurysms [18]. Likewise, a retrospective study including more than 700 patients reported a mean ruptured-aneurysm size below 6 mm [12]. Collectively, these observations indicate that aneurysm diameter alone cannot reliably distinguish stable from rupture-prone lesions and should be interpreted together with anatomical and biological characteristics that are not incorporated into PHASES.

Patient age represents another important aspect of our findings. More than 86% of patients in the present cohort were younger than 70 years and therefore received no age-related points within the PHASES algorithm. This observation illustrates the distinction between the distribution of characteristics among patients who present with rupture and prospective population-based risk prediction. Because the present study contains no unruptured comparison group, these data should not be interpreted as evidence that the age weighting of PHASES is miscalibrated.

Aneurysm location also emerged as an important observation. Although ICA aneurysms represented the largest subgroup, aneurysms located within the anterior communicating artery demonstrated the smallest median diameter while remaining among the most frequent rupture sites. This finding is consistent with the concept that local haemodynamics, vessel geometry, and anatomical location contribute to aneurysm instability beyond size alone. We also observed a significant sex-related difference in aneurysm distribution: AComA aneurysms predominated among men, whereas ICA aneurysms predominated among women. Because this was a ruptured-only single-centre cohort, the sex difference should be regarded as descriptive and cannot establish sex-specific or location-specific rupture risk.

An additional issue concerns patients harbouring multiple aneurysms. Current PHASES recommendations provide no clear guidance regarding rupture-risk estimation in this population, and previous studies have identified aneurysm multiplicity as an unresolved limitation of the score [19]. Because only 14 patients in the present cohort harboured multiple aneurysms, robust subgroup analysis of multiplicity was not possible; nevertheless, this factor remains clinically relevant and warrants further investigation.

Arterial hypertension was documented in 17.70% of the present cohort. Although this proportion is lower than that reported in many international aSAH series, regional Polish data demonstrate substantial variation according to case selection and ascertainment. In a population-based analysis from the Silesian Province covering 2009–2019, a previous history of hypertension was identified in 31.7% of all SAH cases but in only 21% of patients who underwent aneurysm-directed intervention, a value closer to that observed in our treated cohort [20]. In that study, hypertension was identified from prior hospital diagnoses, illustrating how retrospective ascertainment can influence recorded prevalence. Accordingly, under-ascertainment of premorbid hypertension in our retrospective clinical records cannot be excluded. Because hypertension contributes one PHASES point, any such under-ascertainment could shift some patients toward lower estimated-risk categories; the headline distribution of low PHASES estimates should therefore be interpreted with this limitation in mind.

The findings of the present study should not be interpreted as evidence against the clinical usefulness of PHASES. PHASES remains a well-validated and widely implemented risk score [17]. However, our results, together with accumulating evidence from retrospective ruptured-aneurysm cohorts, support its use as one component of clinical assessment rather than as a stand-alone predictor of individual aneurysm rupture.

A possible explanation for these observations is that PHASES intentionally relies on six simple clinical variables and does not incorporate several factors that have emerged as potential determinants of aneurysm instability. Morphological irregularities, daughter sacs, aspect ratio, size ratio, vessel geometry, aneurysm-wall enhancement on vessel-wall magnetic resonance imaging, longitudinal aneurysm growth, haemodynamic characteristics, and emerging biological markers have all been associated with aneurysm instability but remain outside the current PHASES algorithm. Recent computational and statistical studies have demonstrated that aneurysm morphology, including aspect ratio, size ratio, and irregular configuration, contributes information beyond the variables incorporated into PHASES [21]. Future prediction models may benefit from integrating these imaging and biological parameters with established clinical predictors.

Other clinical risk models may complement PHASES in the longitudinal assessment of unruptured intracranial aneurysms. In particular, the ELAPSS score was developed to estimate the 3- and 5-year risk of aneurysm growth rather than rupture itself [22]. Because documented aneurysm growth is an important marker of aneurysm instability, ELAPSS may provide complementary information when interpreted together with PHASES, aneurysm morphology, serial imaging findings, and individual patient characteristics.

The principal strength of the present study lies not in demonstrating that low PHASES scores may precede aneurysm rupture—a finding already reported by previous investigators—but in providing a detailed clinical characterisation of patients in whom rupture occurred despite low predicted risk. Identifying these profiles may support refinement of risk-assessment strategies. Prospective multicentre studies including patients with both ruptured and unruptured aneurysms are required to determine whether integrating morphological and biological markers improves individualised rupture prediction beyond PHASES alone.

### 4.1. Limitations

Several limitations should be acknowledged. First, this retrospective single-centre observational study included only patients presenting with aneurysmal subarachnoid haemorrhage. It therefore describes the distribution of PHASES estimates among ruptured aneurysms but cannot directly assess discrimination, calibration, sensitivity, specificity, or absolute rupture risk in comparison with patients harbouring unruptured aneurysms. A prospective or appropriately matched comparative cohort containing both ruptured and unruptured aneurysms would be required for those purposes. Second, PHASES scores were reconstructed retrospectively from clinical and imaging information available at presentation. Aneurysm dimensions were measured after rupture and therefore may not precisely represent pre-rupture size. Partial thrombosis was assessed during every three-dimensional rotational angiographic examination; however, because three-dimensional rotational angiography primarily depicts the contrast-filled lumen, the true outer diameter may still be underestimated in partially thrombosed lesions. Third, hypertension status was derived from retrospective clinical documentation; previously unrecognised or undocumented hypertension may therefore have been missed and could have lowered the reconstructed PHASES score in some patients. Earlier SAH from another aneurysm was also not available as a dedicated structured variable; in the absence of documentation, no additional PHASES point was assigned, so under-ascertainment of this component cannot be completely excluded. Fourth, several potentially relevant morphological and haemodynamic variables, as well as smoking status and family history, were unavailable and could not be incorporated into the analyses. Fifth, multiple exploratory comparisons were performed without formal adjustment for multiplicity, increasing the possibility of type I error. Finally, the modest number of patients in some subgroups limited the precision of subgroup comparisons.

Future prediction models should move beyond population-based clinical scores by integrating aneurysm morphology, vessel-wall imaging, haemodynamic parameters, artificial intelligence-based image analysis, and longitudinal aneurysm growth. Such multimodal approaches may provide more accurate individualised rupture-risk assessment than currently available clinical scores alone.

### 4.2. Clinical Implications

The present findings support the use of PHASES as an important component of clinical decision-making but emphasise that the score should not be interpreted in isolation. Younger patients with small aneurysms and apparently low PHASES estimates may still harbour lesions capable of rupture. Comprehensive assessment should therefore combine PHASES with aneurysm morphology, imaging characteristics, documented growth, complementary longitudinal tools such as ELAPSS where appropriate, and individual patient factors when determining management.

## 5. Conclusions

Low PHASES-estimated rupture risks were common among patients presenting with ruptured intracranial aneurysms. Younger age, small aneurysm size, and selected aneurysm locations characterised many patients despite apparently reassuring risk estimates. PHASES should be used as a population-based decision-support tool and interpreted alongside aneurysm morphology, imaging findings, documented growth, and patient-specific factors. Prospective comparative studies are required to determine whether multimodal models can improve individualised rupture-risk assessment.

## Figures and Tables

**Table 1:** Baseline demographic, clinical, and aneurysm characteristics of the ruptured intracranial aneurysm cohort (*n* = 243).

Characteristic	Value
Demographic characteristics	
Age, years	56.13 ± 13.10; median 57.0 (range 21–93)
Age < 70 years	210 (86.42%)
Age ≥ 70 years	33 (13.58%)
Female sex	158 (65.02%)
Male sex	85 (34.98%)
Clinical characteristics	
Arterial hypertension	43 (17.70%)
Documented previous subarachnoid haemorrhage	0 (0%)
Aneurysm multiplicity	
One aneurysm	229 (94.24%)
Two aneurysms	13 (5.35%)
Three aneurysms	1 (0.41%)
Aneurysm morphology	
Maximum diameter, mm	7.85 ± 4.76; median 6.50 (range 1.5–30.0)
Maximum diameter ≤ 5 mm	84 (34.57%)
Maximum diameter 5.1–7.0 mm	63 (25.93%)
Maximum diameter 7.1–10.0 mm	52 (21.40%)
Maximum diameter > 10 mm	44 (18.11%)
Aneurysm location	
Internal carotid artery	70 (28.81%)
Anterior communicating artery	65 (26.75%)
Middle cerebral artery	54 (22.22%)
Basilar artery	17 (7.00%)
Other locations	37 (15.23%)
PHASES characteristics	
PHASES score	5.16 ± 3.43; median 4.0 (range 0–16)
PHASES score 0–2	54 (22.22%)
PHASES score 3–5	92 (37.86%)
PHASES score ≥ 6	97 (39.92%)
Estimated 5-year rupture risk, %	2.37 ± 3.46; median 0.9 (range 0.4–17.8)
Estimated risk < 1%	125 (51.44%)
Estimated risk 1–2%	40 (16.46%)
Estimated risk 2–5%	46 (18.93%)
Estimated risk ≥ 5%	32 (13.17%)

Values are presented as *n* (%) unless otherwise indicated. Continuous variables are shown as mean ± standard deviation and median (range). Abbreviations: *n*, number of patients; PHASES, Population, Hypertension, Age, Size, Earlier subarachnoid haemorrhage, and Site.

**Table 2:** Mapping of anatomical labels used in the database to PHASES aneurysm-site categories.

Descriptive Anatomical Label	*n*	PHASES Site Category	PHASES Points
Internal carotid artery (ICA)	70	ICA	0
Middle cerebral artery (MCA)	54	MCA	2
Anterior communicating artery (AComA)	65	ACA/AComA/PComA	4
Basilar artery	17	Posterior circulation	4
Other locations *	37	ACA/AComA/PComA or posterior circulation	4

* The 37 cases reported descriptively as “other locations” comprised ACA sites other than AComA (*n* = 15), PComA (*n* = 5), PCA (*n* = 6), vertebral/PICA sites (*n* = 10), and one additional basilar/other label that mapped to the 4-point PHASES site category (*n* = 1). All 37 therefore received 4 site points. Abbreviations: PHASES, Population, Hypertension, Age, Size, Earlier subarachnoid haemorrhage, and Site; *n*, number of patients; ICA, internal carotid artery; MCA, middle cerebral artery; ACA, anterior cerebral artery; AComA, anterior communicating artery; PComA, posterior communicating artery; PCA, posterior cerebral artery; PICA, posterior inferior cerebellar artery.

**Table 3:** Complete PHASES scoring system and distribution of component variables in the study cohort (*n* = 243).

PHASES Component	Category	Points	Study Cohort, *n* (%)
**Population**	North America/Europe (non-Finnish)	0	243 (100.00%)
	Japan	3	0 (0.00%)
	Finland	5	0 (0.00%)
**Hypertension**	No	0	200 (82.30%)
	Yes	1	43 (17.70%)
**Age**	<70 years	0	210 (86.42%)
	≥70 years	1	33 (13.58%)
**Aneurysm size**	<7.0 mm	0	122 (50.21%)
	7.0–9.9 mm	3	59 (24.28%)
	10.0–19.9 mm	6	51 (20.99%)
	≥20.0 mm	10	11 (4.52%)
Earlier SAH *	No	0	243 (100.00%)
	Yes	1	0 (0.00%)
**Aneurysm site**	ICA	0	70 (28.81%)
	MCA	2	54 (22.22%)
	ACA/AComA/PComA or posterior circulation	4	119 (48.97%)

* Earlier SAH refers to subarachnoid haemorrhage from another aneurysm. Abbreviations: PHASES, Population, Hypertension, Age, Size, Earlier subarachnoid haemorrhage, and Site; *n*, number of patients; SAH, subarachnoid haemorrhage; ICA, internal carotid artery; MCA, middle cerebral artery; ACA, anterior cerebral artery; AComA, anterior communicating artery; PComA, posterior communicating artery.

**Table 4:** Mapping of total PHASES score to estimated 5-year rupture risk.

Total Score	≤2	3	4	5	6	7	8	9	10	11	≥12
5-year risk	0.4%	0.7%	0.9%	1.3%	1.7%	2.4%	3.2%	4.3%	5.3%	7.2%	17.8%

Abbreviations: PHASES, Population, Hypertension, Age, Size, Earlier subarachnoid haemorrhage, and Site.

**Table 5:** Descriptive characteristics according to PHASES-estimated 5-year rupture risk (<1% versus ≥1%).

Characteristic	Risk < 1% (*n* = 125)	Risk ≥ 1% (*n* = 118)
Age, years, mean ± SD	52.95 ± 13.09	59.49 ± 12.30
Age ≥ 70 years	8 (6.40%)	25 (21.19%)
Documented arterial hypertension	9 (7.20%)	34 (28.81%)
Maximum aneurysm diameter, median (IQR), mm	5.0 (4.0–6.0)	10.0 (7.0–13.0)
PHASES size < 7.0 mm	108 (86.40%)	14 (11.86%)
PHASES size 7.0–9.9 mm	17 (13.60%)	42 (35.59%)
PHASES size 10.0–19.9 mm	0 (0%)	51 (43.22%)
PHASES size ≥ 20.0 mm	0 (0%)	11 (9.32%)
PHASES site: ICA (0 points)	50 (40.00%)	20 (16.95%)
PHASES site: MCA (2 points)	26 (20.80%)	28 (23.73%)
PHASES site: ACA/AComA/PComA or posterior circulation (4 points)	49 (39.20%)	70 (59.32%)
PHASES score, median (IQR)	3 (1–4)	8 (6–10)

Values are presented as *n* (%) unless otherwise indicated. This table is descriptive; no *p*-values are reported because the compared variables are components of the PHASES score and therefore contribute directly to the risk-group definition. Abbreviations: *n*, number of patients; SD, standard deviation; PHASES, Population, Hypertension, Age, Size, Earlier subarachnoid haemorrhage, and Site; ICA, internal carotid artery; MCA, middle cerebral artery; ACA, anterior cerebral artery; AComA, anterior communicating artery; PComA, posterior communicating artery; IQR, interquartile range.

## Data Availability

The data presented in this study are not publicly available because they contain confidential clinical information. De-identified data may be made available by the corresponding author upon reasonable request and subject to applicable institutional and ethical approvals.
